# Atezolizumab Immunotherapy-Induced Encephalitis in a Patient With Triple-Negative Breast Cancer: A Case Report

**DOI:** 10.7759/cureus.99387

**Published:** 2025-12-16

**Authors:** Abdul Wahab, Ahmed Faraz

**Affiliations:** 1 Medicine, Hampshire Hospitals NHS Foundation Trust, Basingstoke, GBR; 2 Medicine, Pakistan Air Force Hospital, Islamabad, PAK

**Keywords:** acute encephalitis, anti-gad antibodies, atezolizumab, immunotherapy in breast cancer, intravenous immunoglobulins (ivig), pdl-1 inhibitor

## Abstract

Atezolizumab, a programmed death-ligand 1 inhibitor, is a cornerstone in the management of various advanced malignancies, including triple-negative breast cancer. While generally well-tolerated, it can induce severe immune-related adverse events, with central nervous system (CNS) encephalitis being a rare but potentially life-threatening complication. We present the case of a 33-year-old female patient with stage IV triple-negative breast cancer who developed acute progressive confusion and a fluctuating Glasgow Coma Scale (GCS) following atezolizumab therapy. Diagnostic workup, including brain MRI and EEG, revealed findings consistent with encephalitis, while extensive investigations ruled out infectious, metabolic, and other autoimmune etiologies. The presence of anti-glutamic acid decarboxylase antibodies was noted, suggesting a potential autoimmune predisposition. Prompt multidisciplinary management with high-dose intravenous methylprednisolone, plasmapheresis, and intravenous immunoglobulin led to a gradual and significant improvement in cognitive function and GCS, with no recurrence of encephalitis during follow-up. This case highlights the critical importance of maintaining a high index of suspicion for atezolizumab-induced CNS encephalitis, emphasizing the need for early diagnosis, comprehensive diagnostic evaluation, and aggressive immunosuppressive therapy to achieve favorable neurological outcomes in patients receiving immune checkpoint inhibitors.

## Introduction

Atezolizumab, a programmed death-ligand 1 (PD-L1) inhibitor, has revolutionized the treatment landscape for various advanced malignancies, including triple-negative breast cancer [1-3]. Its mechanism of action involves blocking the PD-1/PD-L1 signaling pathway, thereby enhancing T-cell activity against tumor cells [4,5]. While highly effective in controlling tumor progression, immune checkpoint inhibitors (ICIs) can induce a spectrum of immune-related adverse events (irAEs) due to broad immune system activation [1,5,6].

Among these irAEs, central nervous system (CNS) involvement, particularly encephalitis, is a rare but potentially life-threatening complication [2,7,8]. The overall incidence of neurological irAEs associated with ICIs ranges from 2% to 7% [2,7], with encephalitis specifically noted to occur in approximately 0.8% to 4.2% of patients receiving atezolizumab therapy [1,4,6]. Case reports have documented atezolizumab-induced encephalitis across various cancer types, including breast, lung, and hepatocellular carcinoma, underscoring the importance of early recognition and multidisciplinary management [4,6,9-12]. This case report highlights a rare instance of atezolizumab-induced CNS encephalitis in a patient with stage IV triple-negative breast cancer, emphasizing the critical need for prompt diagnosis and aggressive immunosuppressive interventions to mitigate neurological sequelae.

This case was previously presented as a poster at the ICON Conference as part of the multidisciplinary oncology poster session on May 23, 2025. 

## Case presentation

A 33-year-old woman with a history of stage IV triple-negative breast cancer, diagnosed in November 2022 during pregnancy with metastases to the liver and right axillary lymphadenopathy, was receiving treatment with subcutaneous atezolizumab, tamoxifen, and nab-paclitaxel. She presented to the hospital with complaints of fatigue, acute-onset progressive confusion, and drowsiness. The patient had been receiving atezolizumab for several months, and her neurological symptoms developed gradually a few days after her most recent dose. Her Glasgow Coma Scale (GCS) was noted to be fluctuating between 7 and 9 upon admission. The cognitive decline had a gradual onset, worsening over several days preceding her presentation. The patient denied any history of recent febrile illness or viral prodrome. On physical examination, there were no signs of meningeal irritation, such as neck stiffness.

Investigations

Initial laboratory investigations are summarized in Table 1.

**Table 1:** Summary of Key Laboratory Investigations

Investigation	Result	Reference Range	
White Blood Cell Count	10 × 10⁹/L	4.0–11.0 × 10⁹/L	
Hemoglobin	138 g/L	120–160 g/L	
Neutrophils	7.7 × 10⁹/L	2.0–7.5 × 10⁹/L	
C-reactive Protein (CRP)	<0.1 mg/dL	<0.5 mg/dL	
Procalcitonin	0.7 ng/mL	<0.5 ng/mL	
Alanine Aminotransferase (ALT)	>900 U/L	7–56 U/L	
Albumin	38 g/L	35–50 g/L	
Cortisol	618 nmol/L	171–536 nmol/L	
CSF White Blood Cell Count	6 cells/µL	<5 cells/µL	
CSF Glucose	4.0 mmol/L	2.8–4.4 mmol/L	
CSF Protein	6.1 g/L	1.5–4.5 g/L	

An extensive workup to exclude infectious etiologies was negative, including serology for viral hepatitis, blood cultures, a comprehensive viral screen, and a respiratory BioFire panel. Autoimmune screening revealed positive anti-glutamic acid decarboxylase (anti-GAD) antibodies. PD-L1 expression was positive. Other autoimmune and paraneoplastic markers, including ANCA, CASPR2, NMDA, and LGI1, were negative.

**Figure 1:**
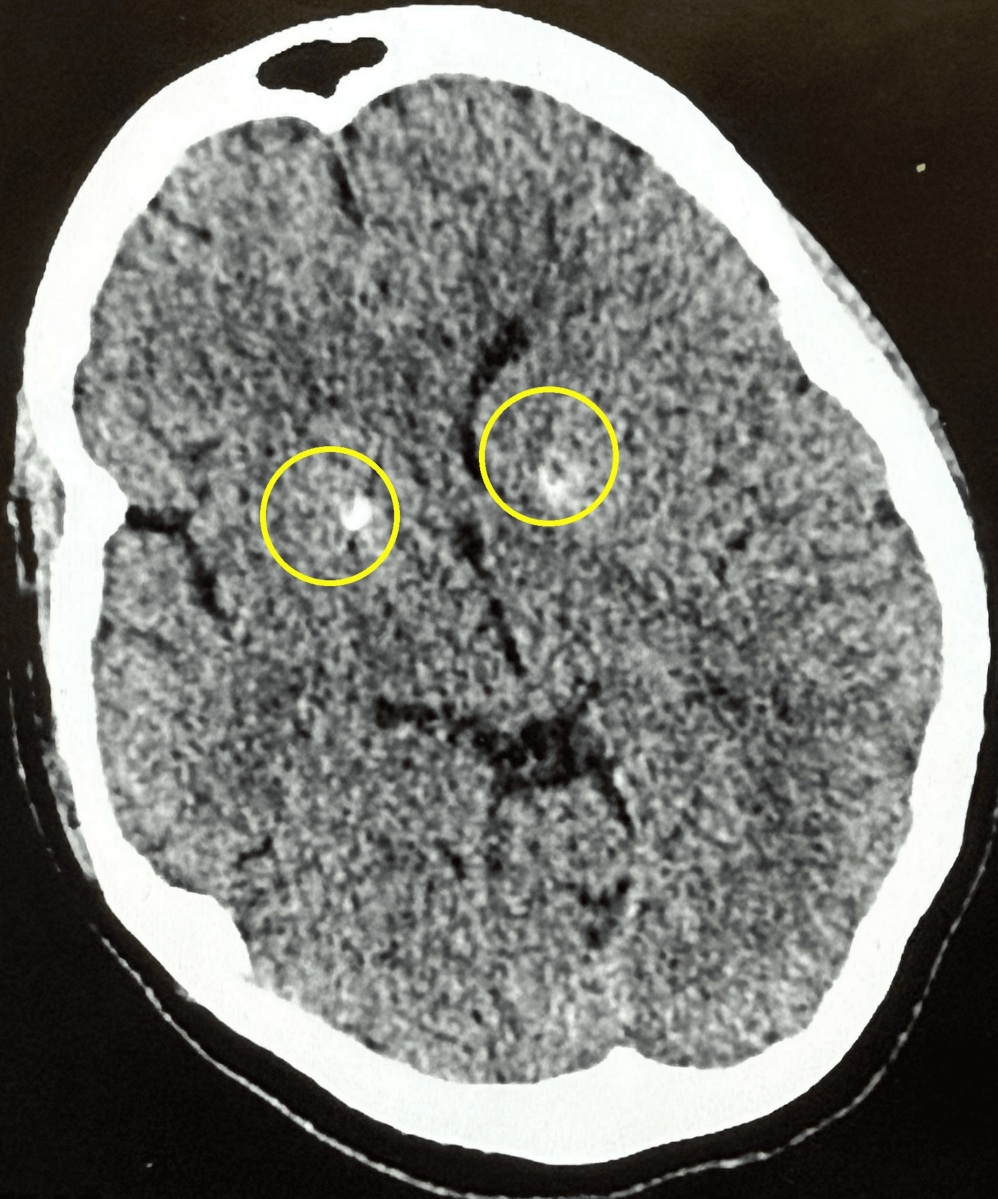
Non-contrast CT Brain Non-contrast CT scan of the brain demonstrating bilateral basal ganglia calcifications, indicated by yellow circles. No evidence of acute intracranial hemorrhage, mass effect, or space-occupying lesion is observed.

**Figure 2:**
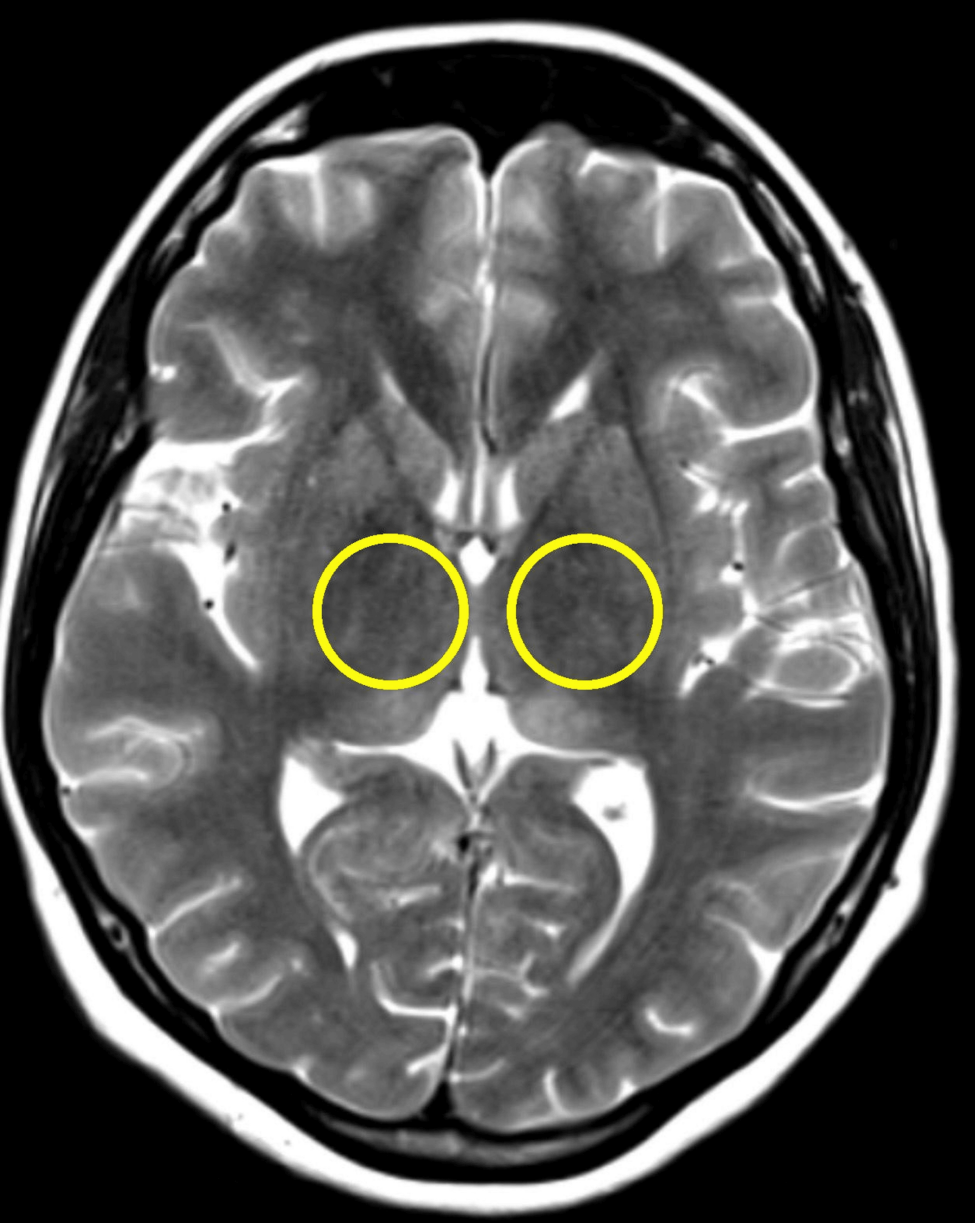
Axial T2-Weighted MRI Brain Image Axial T2-weighted MRI demonstrating bilateral thalamic hyperintensity, more prominent on T2 sequences. These findings are consistent with inflammatory or autoimmune encephalitis and align with reported imaging patterns in immune checkpoint inhibitor–related neurotoxicity.

**Figure 3:**
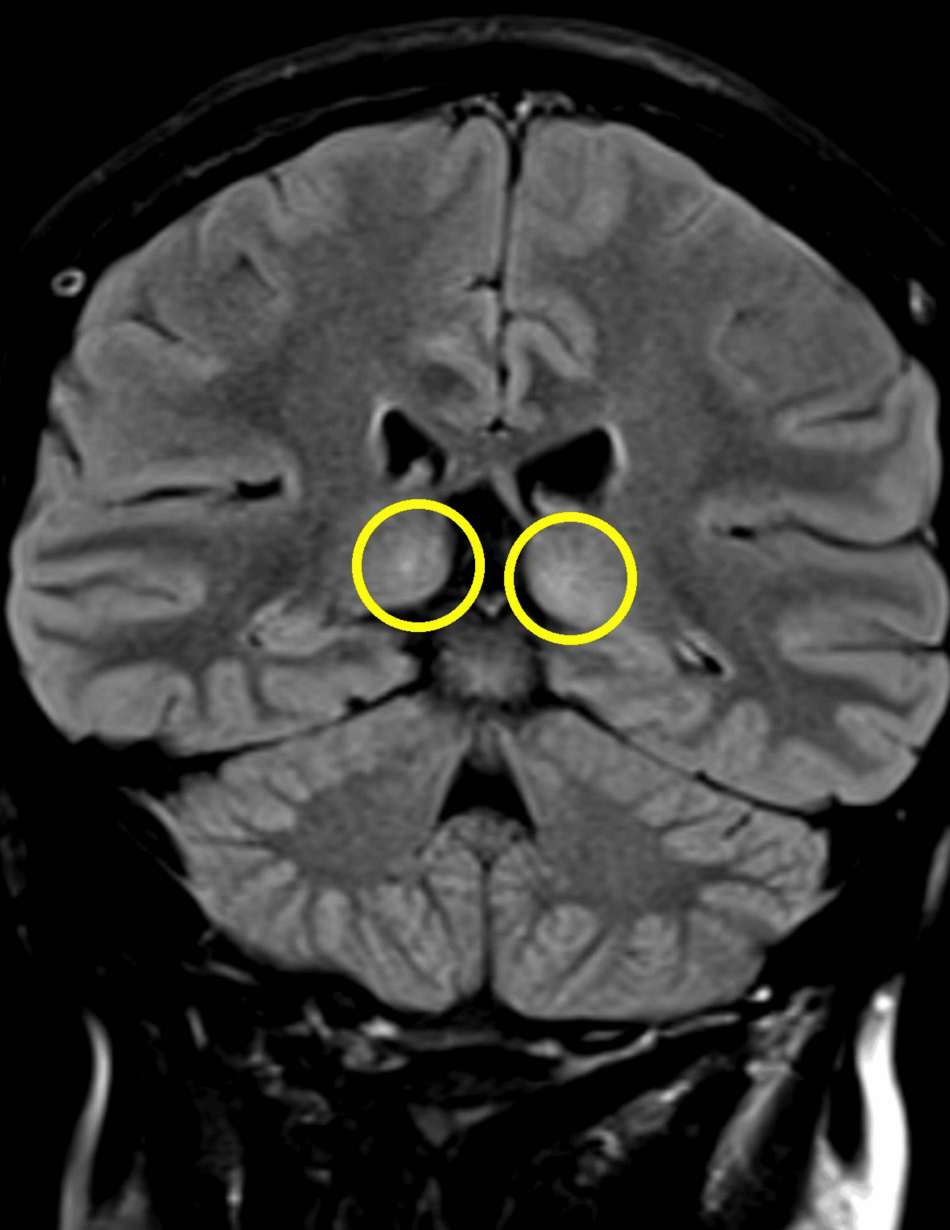
Coronal FLAIR MRI Brain Image Coronal FLAIR MRI sequence showing bilateral thalamic hyperintensity, with increased signal intensity involving the medial thalami. These changes support the diagnosis of atezolizumab-induced encephalitis. FLAIR: Fluid-Attenuated Inversion Recovery

Computed tomography (CT) of the brain revealed bilateral basal ganglia calcification but no evidence of acute intracranial hemorrhage or mass lesions (Figure 1). A subsequent magnetic resonance imaging (MRI) of the brain with contrast administration demonstrated bilateral thalamic hyperintensities on T2-weighted imaging (Figure 2) and fluid-attenuated inversion recovery (FLAIR) sequences (Figure 3), consistent with encephalitis and without leptomeningeal enhancement or metastatic disease. Cerebrospinal fluid (CSF) analysis was unremarkable for infection, and CSF autoantibodies were negative. Electroencephalography (EEG) demonstrated diffuse slowing, compatible with encephalitis.

Multidisciplinary team input

The rheumatology team was consulted to evaluate for a primary rheumatological autoimmune etiology. They concluded that the clinical picture, radiological findings, and negative connective tissue screen did not support a diagnosis of a primary connective tissue disease or an ANCA-associated vasculitis.

The psychiatry team was also involved to rule out the possibility of a late-onset post-partum psychosis, given a single reported episode of auditory hallucinations. They determined that the patient's symptoms were more consistent with an organic brain syndrome secondary to encephalitis and that the features were not typical of post-partum psychosis.

Management and outcome

Based on the clinical presentation, MRI findings, and the exclusion of other potential etiologies, a diagnosis of atezolizumab-induced CNS encephalitis was established. The patient was promptly initiated on high-dose intravenous methylprednisolone to suppress the neuroinflammation.

Due to the severity of her presentation, she also underwent a course of plasmapheresis (PLEX), receiving a total of four sessions. Following the fourth session of PLEX, a notable improvement in her neurological status was observed, although she continued to exhibit significant aphasia and provided inconsistent responses to questions. To further modulate the immune response, she was also treated with intravenous immunoglobulin (IVIG).

A gradual weaning of corticosteroids was planned following her clinical improvement. The patient demonstrated a progressive recovery of her cognitive function and her GCS improved. At follow-up, there were no recurrent episodes of encephalitis.

## Discussion

ICIs, such as atezolizumab, have transformed cancer therapy by enhancing anti-tumor immune responses. Atezolizumab, a PD-L1 inhibitor, blocks the interaction between PD-L1 on tumor cells and PD-1 on T-cells, thereby restoring T-cell-mediated cytotoxicity [1,4]. Although ICIs provide substantial clinical benefit across multiple malignancies, including triple-negative breast cancer, they can precipitate a wide range of irAEs affecting almost any organ system [1,5,6]. Neurological irAEs, although uncommon, are clinically significant due to their potential severity and diverse presentations [1,2,7].

Pathogenesis of ICI-induced encephalitis

The precise mechanisms underlying ICI-induced encephalitis remain uncertain. Current evidence suggests that checkpoint inhibition disrupts immune tolerance, leading to an exaggerated T-cell response against CNS antigens [2,5]. Autopsy studies and case series have demonstrated infiltration of CD8+ T-cells and inflammatory cytokines within the CNS, supporting an immune-mediated process [2,3].

The presence of anti-GAD antibodies in our patient may indicate an underlying autoimmune predisposition, which could have been unmasked or amplified by atezolizumab therapy. This is consistent with reports suggesting that ICIs may trigger or worsen latent autoimmune conditions [1,2].

Clinical manifestations and onset

The clinical presentation of atezolizumab-induced encephalitis is heterogeneous and may mimic various neurological disorders, making early recognition challenging [1,2]. Common symptoms include confusion, disorientation, reduced consciousness, seizures, and focal neurological deficits [1,4]. Symptoms typically occur weeks to months after initiating treatment, although delayed presentations are documented [1,4].

A systematic review reported a median onset of approximately two weeks after the most recent atezolizumab dose [1]. Our patient’s progressive confusion and declining GCS align with previously reported cases. Some reports also describe atypical features such as hypothermia-suggesting hypothalamic involvement-though this was not present in our patient [4]. Other manifestations include high fever, aphasia, dysarthria, and convulsions [4,6,7].

Diagnostic approach

A comprehensive diagnostic evaluation is essential to exclude infectious, metabolic, paraneoplastic, and structural causes of encephalopathy in patients receiving ICIs [2,4]. MRI is the most sensitive neuroimaging tool, often demonstrating hyperintense lesions in cortical, thalamic, or brainstem regions consistent with immune-mediated inflammation [2,4].

In our case, the initial CT brain scan showed only bilateral basal ganglia calcification, whereas subsequent MRI revealed bilateral thalamic hyperintensities consistent with encephalitis. Some published cases describe diffuse leptomeningeal enhancement or reversible splenial lesions characteristic of MERS [6,7].

CSF analysis typically demonstrates elevated protein and lymphocytic pleocytosis, although findings may vary. Markedly elevated CSF protein levels, sometimes exceeding 800 mg/dL, have been reported in severe cases [3]. Our patient’s CSF showed elevated protein with normal cell counts, supporting an autoimmune process.

EEG commonly shows diffuse slowing in cases of ICI-associated encephalitis [1,2]. Multidisciplinary assessment, including rheumatology and psychiatry input, as in our case, helps narrow the differential diagnosis and exclude alternative causes.

Management strategies

Prompt initiation of immunosuppressive therapy is crucial to prevent irreversible neurological injury [1,2,4]. High-dose corticosteroids are considered first-line treatment. For severe or steroid-refractory cases, additional therapies such as IVIG and plasmapheresis are effective adjuncts [2-4].

Our patient received high-dose intravenous methylprednisolone, four sessions of plasmapheresis, and IVIG, resulting in substantial neurological improvement. This treatment response is consistent with favorable outcomes reported in the literature when early intervention is initiated [1,2].

However, full neurological recovery is not universal. Some cases report persistent deficits-including paralysis and aphasia-despite appropriate treatment, particularly when therapy is delayed [6]. Decisions regarding re-initiation of ICI therapy after an irAE must be individualized, balancing oncological benefit against risk of recurrence. Long-term follow-up is essential, as relapses may occur, especially if immunotherapy is resumed [3].

Comparison with existing literature

This case shares several features with previously reported instances of atezolizumab-induced encephalitis, including acute neurological decline and favorable response to immunosuppressive therapy [4,6,7]. Although triple-negative breast cancer is not the most commonly reported malignancy associated with this irAE, atezolizumab-related neurotoxicity has been documented across multiple tumor types [2,10,11].

The detection of anti-GAD antibodies suggests a possible autoimmune predisposition, highlighting the need for further research into biomarkers that might help predict susceptibility to neurological irAEs.

The involvement of rheumatology and psychiatry in our case underscores the importance of a multidisciplinary approach. Their evaluations helped exclude alternative rheumatological and psychiatric diagnoses, strengthening the conclusion of atezolizumab-induced encephalitis.

The complete recovery observed in our patient following aggressive multimodal treatment reinforces the critical value of early recognition and prompt immunosuppression. Given the potential for profound neurological morbidity, clinician awareness and timely intervention remain essential.

## Conclusions

This case report underscores the critical importance of recognizing atezolizumab-induced CNS encephalitis as a rare but severe irAE in patients undergoing immunotherapy for advanced malignancies. Our patient, a 33-year-old woman with stage IV triple-negative breast cancer, developed acute progressive confusion and a reduced GCS following atezolizumab therapy. The diagnosis was established through a meticulous exclusion of infectious, metabolic, and other autoimmune etiologies, supported by characteristic MRI findings and EEG abnormalities. The presence of anti-GAD antibodies further hints at a potential autoimmune predisposition exacerbated by ICI therapy.

Prompt and aggressive multidisciplinary management, involving high-dose intravenous corticosteroids, plasmapheresis, and intravenous immunoglobulin, proved instrumental in achieving a favorable neurological outcome with a gradual improvement in cognitive function and no recurrent episodes of encephalitis. This case highlights the variable clinical presentation of ICI-induced neurotoxicity and emphasizes the necessity for a high index of suspicion among clinicians. Early diagnosis and timely, comprehensive immunosuppressive intervention are paramount to mitigate potentially devastating neurological sequelae and improve patient prognosis. Continued research into predictive biomarkers and refined management protocols for ICI-induced irAEs is essential to optimize patient safety and treatment efficacy in the evolving landscape of cancer immunotherapy.
